# Comparison of retroperitoneal laparoscopic versus open adrenalectomy for large pheochromocytoma: a single-center retrospective study

**DOI:** 10.1186/s12957-019-1649-x

**Published:** 2019-06-29

**Authors:** Wei Zhu, Shaogang Wang, Guanghui Du, Hailang Liu, Jinjin Lu, Weimin Yang

**Affiliations:** Department of Urology, Tongji Hospital, Tongji Medical College, Huazhong University of Science and Technology, 1095#, Jie-Fang Avenue, Qiaokou District, Wuhan, 430030 Hubei China

**Keywords:** Pheochromocytoma, Perioperative outcomes, Open adrenalectomy, Laparoscopy

## Abstract

**Background:**

It remains unclear whether retroperitoneal laparoscopic adrenalectomy (RLA) is safe and effective for the treatment of large pheochromocytoma (PHEO). This retrospective study aimed to identify the advantages and disadvantages of RLA compared to open adrenalectomy (OA).

**Methods:**

This study included 147 patients who underwent RLA (*n* = 101) or OA (*n* = 46) for PHEO larger than 5 cm. Groups were balanced by propensity score matching (PSM) into 46 pairs. Perioperative variables and long-term follow-up results were compared between the two groups.

**Results:**

After PSM, patients in the RLA group had a shorter operative time (218 vs. 245 min, *P* = 0.040), quicker bowel recovery (2 vs. 3 days, *P* = 0.046), and a shorter hospital stay (8 vs. 9 days, *P* = 0.010) compared to the OA group. The results of multiple linear regression analyses showed that the operative method (OA vs. RLA) had an influence on the above three postoperative variables (*β* = 31.84, *P* = 0.046; *β* = 0.76, *P* = 0.044; and *β* = 1.25, *P* = 0.025, respectively). There was no significant difference in the proportion of patients with improved blood pressure (82.61% vs. 69.57%, *P* = 0.143) between the two groups.

**Conclusions:**

Both RLA and OA provide similar perioperative and long-term outcomes for the surgical management of large PHEO. RLA is an efficacious and safe surgical method for patients with PHEO larger than 5 cm in diameter.

## Background

Pheochromocytomas (PHEOs) are rare but dangerous neuroendocrine tumors, approximately 80–85% of which arise from the adrenal medulla [1]. The development of hypertension due to the oversecretion of catecholamines represents one of the most important clinical symptoms of PHEO. In the outpatient department, 0.1–0.6% of patients with hypertension are found to suffer from PHEO [2, 3]. In patients with PHEO, the presentation of hypertensive encephalopathy, cerebrovascular accident, or neurogenic pulmonary edema is always associated with cardiovascular complications, such as sudden death and heart failure, which leads to a poor prognosis [4]. Surgical resection can not only cure hypertension, but also prevent the cardiovascular complications caused by hypertension. Thus, surgical resection is considered the gold standard for the management of PHEO [5].

Surgery for PHEO is challenging because of the release of catecholamines during the operation. For decades, open adrenalectomy (OA) has been the first choice for the surgical management of benign adrenal tumors. However, since the first laparoscopic adrenalectomy reported by Gagner et al. [6], the use of laparoscopic adrenalectomy has gained popularity, especially for the surgical treatment of small- to medium-sized benign tumors [7]. In the resection of PHEO, laparoscopic adrenalectomy has been associated with lower estimated blood loss, shorter hospital stay, and fewer episodes of intraoperative hypertension or hypotension when compared to OA [8–10]. Due to the restricted operating area, laparoscopic adrenalectomy is always chosen for adrenal tumors that are smaller than 7 cm, while OA is still considered the first-line therapy for larger tumors [11, 12]. Conzo et al. [13] claimed that laparoscopic surgery could be safely performed for PHEO larger than 6 cm. On the other hand, OA was strongly recommended as the preferred surgical procedure in the 2014 guidelines of the Endocrine Society, as this procedure ensures that the tumor is completely resected [14]. However, at present, there are not enough comparative studies of the two surgical methods for the treatment of large PHEOs [14, 15].

For large PHEOs, often defined as tumors larger than 5 cm in diameter, the safety and effectiveness of laparoscopic adrenalectomy has been demonstrated. A propensity score-matched cohort study recently performed by Bai et al. [16] reported that the perioperative outcomes of patients with large PHEO who underwent transperitoneal laparoscopic adrenalectomy were significantly better than those who underwent open surgery. Moreover, it has been reported that retroperitoneal laparoscopic adrenalectomy (RLA) is safer, faster, and more feasible than the transperitoneal approach for the treatment of large PHEO [17, 18]. To date, no studies have investigated the oncological outcomes and recurrence rate after OA compared to RLA. Therefore, the aim of the present study was to compare the perioperative outcomes of large PHEO (> 5 cm in diameter) between the two surgical methods.

## Methods

In this retrospective study, we reviewed and compared the results of patients admitted to the Huazhong University of Science and Technology-affiliated Tongji Hospital who underwent RLA or OA for large PHEO between January 2008 and October 2018. The present study was approved by the institutional review board. All cases included in the cohort were confirmed as PHEO by postoperative pathological examination. In our institution, generally, RLA is the first choice for PHEO patients when the tumors smaller than 6 cm. However, it should be noted that sometimes patients choose RLA, although the tumors are larger than 6 cm. Operative informed consent was provided for all patients, and a detailed explanation of the benefits and potential risks of the proposed operative methods was provided. Only patients who underwent RLA or OA for PHEO were included in our analysis.

The RLA method was performed according to the paper reported by Zhang et al. [19]. Under general anesthesia, the RLA was started with a skin incision at the tip of the 12th rib. And the retroperitoneal space was created by balloon dilation. Then, three trocars (i.e., 5-, 10-, and 12-mm trocars) were introduced into the retroperitoneal space. Keep the carbon dioxide pressure of pneumoperitoneum around 25 mmHg. The first dissection plane is the space between the perirenal fat sac located above the kidney and the anterior Gerota fascia. Blunt segregation predominates until the adrenal gland or the anterior surface of the tumor is found. The second dissection plane is between the perirenal fat and the posterior renal fascia located on the lateral side of the upper kidney pole. Third plane dissection progresses immediately adjacent to the parenchymal surface of the upper kidney pole. The last step is to find the central adrenal vein, then clip and transect it. At the same time, the remaining upper adrenal artery and its surrounding adipose tissue were completely transected. The isolated adrenal tumor is placed in a sack and retrieved through the posterior-axillary trocar port site. The surgery was performed by four experienced surgeons in our center, one of whom was recognized as the pioneer in laparoscopy and supervised the quality of surgery. As for the OA method, patients lie on the operating table and the appropriate incision length was chosen according to the size of the tumor. Firstly, the right lobe of the liver was pushed aside when resecting the right tumors, the spleen and pancreas were pushed aside when resecting the left tumors, and the tissue structure around the tumors was dissected. Subsequently, the adrenal vein was exposed at the inferior margin of the left adrenal or the medial margin of the right adrenal, and then double ligation of the adrenal vein was performed. After the tumors were completely separated, the whole specimen was taken out.

Initially, we excluded those patients with extra-adrenal PHEO or paraganglioma according to their medical history or imaging data. To ensure the accuracy of surgical data, patients with multiple tumors in one adrenal gland were excluded from the study. Those with a lesion ≤ 5 cm on the preoperative imaging examination were also excluded. Moreover, using the intention-to-treat principle, RLA-converted-to-OA cases were considered RLA in the present study. All remaining patients (i.e., those with a single tumor > 5 cm) who underwent RLA or OA in our hospital were included in the present study.

Propensity score matching (PSM) was used to adjust for differences between the two groups, which helps to make the results as accurate as possible. The propensity score for each patient was determined by multivariate logistic regression based on tumor size and tumor laterality, where the caliper width for the PSM was set as 0.1.

Relevant clinical data including demographics, laboratory examination, imaging data, operative details, hospital stay, follow-up results, and postoperative complications were recorded and compared between PHEO patients who underwent RLA and OA. Basic demographic information including the age, gender, body mass index (BMI), American Society of Anesthesiologists Physical Status Classification System (ASA) score, tumor size, and tumor side of all patients was initially analyzed between the two groups. Perioperative indicators, including both intraoperative and postoperative terms, were compared to evaluate the efficacies of the two procedures, which included the operation time, estimated blood loss, number of intraoperative transfusions, number of instances of hemodynamic instability (HI) (systolic blood pressure > 180 mmHg or mean arterial pressure < 60 mmHg) [20], number of complications, length of hospitalization, duration of draining, and time to resume completely normal diet. Evaluation of the long-term effects consisted of the number of improvement in blood pressure (BP). Improvement BP was defined as a decrease in BP compared to that before surgery or a decrease in the number of antihypertensive drugs.

Statistical Product and Service Solutions version 24.0 (IBM Corporation, Armonk, NY, USA) software was used to conduct all analyses in the present study. Continuous variables with normal distributions were expressed as mean ± standard deviation (SD). Differences between groups were determined using the independent sample Student’s *t* test for variables with normal distributions. The results for continuous variables without normal distribution were demonstrated as median (range) and assessed by the Mann-Whitney *U* test. Categorical variables were expressed as frequencies and analyzed with the chi-square test or Fisher’s exact test. The odds ratio (OR) and *β* coefficient were determined by binary logistic regression and multiple linear regression, respectively, and the 95% confidence interval (CI) was also calculated. Importantly, a Box-Cox transformation was applied to the variable without normal distribution before multiple linear regression explained to achieve normal conditions. In this present study, a two-tailed *P* value of < 0.05 was considered statistically significant.

## Results

The basic clinical features of patients are summarized in Table 1. All patients who met the inclusion criteria were divided into RLA (*n* = 101) and OA (*n* = 46) groups. It should be noted that 6 (5.94%) cases in the RLA group required conversion to the open approach. Conversion to open surgery was necessary in two cases for uncontrollable bleeding and four cases for technical constraints. After PSM, the unbalanced differences between the two groups were removed, and the 147 patients were matched into 46 pairs.

**Table 1:** The basic demographics and pre-operative features of included patients

	Before PSM			After PSM		
Variable	RLA (*n* = 101)	OA (*n* = 46)	*P* value	RLA (*n* = 46)	OA (*n* = 46)	*P* value
Age (years)	47.15 ± 12.96	45.17 ± 10.29	0.364	48.15 ± 12.68	45.17 ± 10.29	0.219
Male	48 (47.52)	28 (60.87)	0.123	24 (52.17)	28 (60.87)	0.400
Body mass index (kg × m^−2^)	21.98 ± 3.04	21.67 ± 2.73	0.550	21.62 ± 2.70	21.67 ± 2.73	0.934
Hypertension	66 (65.35)	32 (69.57)	0.856	29 (63.04)	32 (69.57)	0.508
VMA (μmol/24 h)	96.75 (14.20, 689.60)	110.20 (14.55, 371.48)	0.966	119.00 (43.53, 340.58)	110.20 (14.55, 371.48)	0.372
eGFR (ml/min/1.73 m^2^)	101.1 (62.2, 145.1)	102.8 (71.8, 140.2)	0.768	96.7 (73.3, 132.7)	102.8 (71.8, 140.2)	0.705
Preoperative hemoglobin (g/L)	131 (71, 164)	127 (72, 161)	0.851	122 (80, 149)	127 (72, 161)	0.291
Comorbidities [*n* (%)]						
Myocardial infarction	3 (2.97)	0 (0)	0.552	3 (6.52)	0 (0)	0.242
Stroke	4 (3.96)	3 (6.52)	0.678	2 (4.35)	3 (6.52)	0.646
Coronary heart disease	9 (8.91)	2 (4.35)	0.330	4 (8.70)	2 (4.35)	0.398
Diabetes	18 (17.82)	10 (21.74)	0.575	9 (19.57)	10 (21.74)	0.797
ASA score [*n* (%)]						
III/IV	29 (28.71)	10 (21.74)	0.375	12 (26.09)	10 (21.74)	0.625
Tumor size (cm) (range)	6.48 ± 1.83 (5.1–9.8)	7.92 ± 1.98 (5.1–13.2)	< 0.001	7.76 ± 2.02 (5.1 9.4)	7.92 ± 1.98 (5.1–13.2)	0.704
Tumor laterality [*n* (%)]						
Bilateral	4 (3.96)	2 (4.35)	0.856	2 (4.35)	2 (4.35)	0.975
Right	57 (56.44)	28 (60.87)		29 (63.04)	28 (60.87)	
Left	40 (39.60)	16 (34.78)		15 (32.61)	16 (34.78)	

*Abbreviations*: *PSM* the propensity score matching, *RLA* retroperitoneal laparoscopic adrenalectomy, *OA* open adrenalectomy, *BP* blood pressure, *VMA* vanillic mandelic acid, *eGFR* estimated glomerular filtration rate, *ASA* American Society of Anesthesiologists Physical Status Classification System

Tumor size was the only unbalanced variable between the two groups before PSM, as tumors were significantly bigger in the OA group than in the RLA group (7.92 ± 1.98 vs. 6.48 ± 1.83 cm, *P* < 0.001). After PSM, the mean tumor size was 7.76 ± 2.02 cm and 7.92 ± 1.98 cm in the RLA and OA groups (*P* = 0.704), respectively, and there was no longer any statistically significant difference. Except for tumor size, the other baseline variables including age, BMI, history of hypertension, comorbidities, ASA score, and preoperative laboratory examinations were not significantly different between the two groups before and after PSM (Table 1). After adjusting the unbalanced variable, the mean age of patients in the OA group was 45.17 ± 10.29 years and in the RLA group was 48.15 ± 12.68 years (*P* = 0.219). Most patients in the RLA and OA groups also had hypertension (63.04% vs. 69.57%, *P* = 0.508) and a low ASA score (< III; 73.91% vs. 78.26%, *P* = 0.625).

The perioperative outcomes are summarized in Table 2. The incidence of HI during surgery, intraoperative blood transfusion, and postoperative complications in the RLA group were similar to the OA group (54.35% vs. 56.52%, *P* = 0.834; 45.65% vs. 63.04%, *P* = 0.094; and 45.65% vs. 34.78%, *P* = 0.288, respectively). Moreover, the difference in estimated blood loss between the two groups (200 vs. 500 ml, *P* = 0.794) was not statistically significant. Nevertheless, it is necessary to emphasize that OA is associated with 3 times more blood loss. However, another noteworthy result is that the incidence of intraoperative transfusion was higher in the OA group compared to the RLA group (63.04% vs. 45.65%). Although these differences were not statistically significant (*P* = 0.094), we could speculate that intraoperative blood transfusion was needed when massive intraoperative bleeding occurs which may keep hemoglobin from fluctuating violently. Moreover, there was a shorter operative time, quicker bowel recovery, and shorter length of hospital stay in the RLA group (218 vs. 245 min, *P* = 0.040; 2 vs. 3 days, *P* = 0.046; and 8 vs. 9 days, *P* = 0.010) compared to the OA group. The results of long-term follow-up are also shown in Table 2. There was no significant difference in the proportion of patients with improved BP between the two groups (82.61% vs. 69.57%, *P* = 0.143).

**Table 2:** The perioperative and follow-up outcomes of included patients

	Before PSM			After PSM		
Variable	RLA (*n* = 101)	OA (*n* = 46)	*P* value	RLA (*n* = 46)	OA (*n* = 46)	*P* value
Intraoperative data						
Hemodynamic instability [*n* (%)]	46 (45.54)	26 (56.52)	0.217	25 (54.35)	26 (56.52)	0.834
Operating time (min)	185 (89, 520)	245 (107, 480)	0.003	218 (93, 367)	245 (107, 480)	0.040
Estimated blood loss (mL)	150 (50, 2300)	500 (50, 3000)	0.047	200 (50, 1500)	500 (50, 3000)	0.794
Transfusion [*n* (%)]	31 (30.69)	29 (63.04)	< 0.001	21 (45.65)	29 (63.04)	0.094
Immediate postoperative hemoglobin (g/L)	106 (69, 161)	107 (79, 150)	0.607	107 (77,138)	107 (79, 150)	0.721
Change in hemoglobin (g/L)	18.0 (7.0, 38.0)	10.0 (5.0, 28.0)	0.206	14.0 (7.0, 27.3)	10.0 (5.0, 28.0)	0.412
Open conversion [*n* (%)]	6 (5.94)	–	–	2 (4.35)	–	–
Postoperative data						
Postoperative complications [*n* (%)]						
Overall morbidity	39 (38.61)	16 (34.78)	0.656	21 (45.65)	16 (34.78)	0.288
Clavien III/IV	15 (14.85)	4 (8.70)	0.489	7 (15.22)	4 (8.70)	0.443
Cardiovascular morbidity causes [*n* (%)]						
Postoperative hypertension	12 (11.88)	5 (10.87)	0.859	8 (17.39)	5 (10.87)	0.369
Postoperative transfusion	9 (8.91)	0 (0)	0.057	4 (8.70)	0 (0)	0.058
Postoperative hypotension	8 (7.92)	3 (6.52)	0.765	5 (10.87)	3 (6.52)	0.459
Return to diet (days)	2 (1, 3)	3 (1, 4)	0.003	2 (1, 2)	3 (1, 4)	0.046
Time to drain removal (days)	4 (2, 8)	4 (3, 8)	0.997	4 (2, 7)	4 (3, 8)	0.174
Postoperative hospital stay (days)	7 (4, 12)	9 (5, 14)	< 0.001	8 (5, 10)	9 (5, 14)	0.010
Follow-up data						
Follow-up period (months)	42.6 (2.8, 118.8)	70.8 (4.0, 117.5)		40.9 (9.5, 102.5)	70.8 (4.0, 117.5)	
Improvement BP [*n* (%)]	81 (80.20)	32 (69.57)	0.156	38 (82.61)	32 (69.57)	0.143

*Abbreviations*: *PSM* the propensity score matching, *RLA* retroperitoneal laparoscopic adrenalectomy, *OA* open adrenalectomy, *bpm* beats per minute, *MAP* mean arterial pressure, *SBP* systolic blood pressure, *HR* heart rate

Table 3 presents the results from binary logistic regression and multiple linear regression analyses to determine the potential influence of surgical methods on the perioperative outcome. The results of multiple linear regression analyses showed that the operative method (OA vs. RLA) influenced the operation time (*β* = 31.84, *P* = 0.046), bowel recovery (*β* = 0.76, *P* = 0.044), and postoperative hospital stay (*β* = 1.25, *P* = 0.025), while logistic regression analysis showed no positive correlation between the operative method and perioperative outcomes.

**Table 3:** The results from binary logistic regression and multiple linear regression analyses of included patients

	Before PSM			After PSM		
Variable	OR/*β*	95% CI	*P* value	OR/*β*	95% CI	*P* value
Intraoperative HI^#^	1.02	0.46–2.39	0.899	1.07	0.42–2.72	0.888
Intraoperative transfusion^#^	2.61	1.10–6.21	0.021	2.20	0.85–5.55	0.098
Operation time^&^	37.40	8.90–66.59	0.012	31.84	3.35–67.00	0.046
Estimated blood loss^&^	151.16	− 268.05 to 570.42	0.476	55.81	− 532.54 to 644.18	0.850
Postoperative complications^#^	0.70	0.22–1.69	0.442	0.60	0.24–1.48	0.273
Drainage time^&^	− 0.58	− 1.22 to 0.25	0.158	0.24	− 0.08 to 0.61	0.151
Return to diet^&^	0.35	0.02–0.58	0.049	0.76	0.06–1.58	0.044
Postoperative hospital stay^&^	1.38	0.42–2.38	0.005	1.25	0.15–2.33	0.025

*Abbreviations*: *PSM* the propensity score matching, *HI* hemodynamic instability, *OR* odds ratio, *CI* confidence interval

^#^Binary logistic regression

^&^Multiple linear regression

## Discussion

The location and anatomical structure of the adrenal gland make adrenal surgery one of the most high-risk urologic operations. Adrenalectomy for PHEO is more challenging than for other adrenal tumors due to the higher catecholamine excretion and richer vascularity in this kind of tumor, especially in larger ones. Over the past decades, OA has been the standard surgical treatment for PHEO, which leaves a large surgical incision even for small tumors [21]. With the development of laparoscopic technology, many minimally invasive adrenalectomy methods for adrenal tumors have emerged, one of which is posterior retroperitoneoscopic adrenalectomy, described by Mercan et al. [22]. Advantages and disadvantages have been reported for both OA and RLA. Compared to RLA, OA has better intraoperative visibility and a larger workspace, but this is accompanied by a larger surgical incision [23]. Moreover, laparoscopic adrenalectomy is particularly difficult for patients who have ever undergone abdominal surgery. Some researchers have compared the safety and effectiveness of laparoscopic adrenalectomy with OA, showing that endoscopic approaches (conventional laparoscopic, retroperitoneoscopic, or robotic-assisted) have a similar incidence of complications, decreased blood loss during the operation, and a shorter hospital stay after the operation when compared to OA [9, 24–26]. However, only a few studies have focused on the differences in unbalanced variables, such as tumor size, when comparing different surgical methods, which are believed to be associated with perioperative outcomes [27].

In the present study, we reviewed almost 10 years of experience in the surgical treatment of PHEOs larger than 5 cm, including perioperative and long-term follow-up results, and used PSM to remove unbalanced baseline differences so that we could obtain more reliable conclusions. After PSM, the results indicate that RLA is superior to OA in terms of operation time, length of hospital stay, and bowel recovery time. Since Zhang et al. [19] promoted standardized RLA, this method has been widely used in China. In our institution, RLA has been the preferred surgical procedure for adrenal tumors, including PHEO, for many years. Consistent with a previous report [28], the operation time for RLA was found to be shorter than that for OA. This could be explained by the skilled use of laparoscopy by experienced surgeons, which partly offsets the limitation of restricted operating space when resecting PHEO. During OA, abdominal structures and organs are disturbed to a greater extent, which can lead to severe ischemia-reperfusion injury and organ dysfunction after operation. This may explain why those patients undergoing OA required more time to return to an oral diet and had a longer hospital stay. Furthermore, the results of the multiple linear regression analysis confirmed the above findings.

Intraoperative HI and postoperative complications are critical to the postoperative recovery of patients. The incidence of intraoperative HI during adrenalectomy for PHEO has been reported to range between 17–83% [29], which is in accordance with our results. However, neither component comparison nor logistic regression found any correlation between surgical modalities and HI. Similarly, no differences were found when the incidence of postoperative complications was compared between the RLA and OA groups. As mentioned above, RLA is more difficult to perform than OA. Due to the limited visual field and operating space, unexpected disruption of tumors during RLA may cause severe HI, and intraoperative bleeding is more difficult to manage when this occurs. More importantly, these results suggest that the perioperative outcomes of RLA are similar to those of OA in the surgical treatment of PHEO. Long-term follow-up results indicated a comparable improvement rate in BP between the two groups. As we mentioned above, laparoscopic adrenalectomy has been widely used in recent years even for those larger PHEOs. Before that, OA was still the first choice. And this may lead to significant differences in the follow-up period between the two groups.

At present, laparoscopic adrenalectomy, which can be divided into the transperitoneal approach and the retroperitoneal approach, has become the standard treatment for benign adrenal tumors and is recommended in the clinical practice guideline [14]. Conzo et al. [13] and Shiraishi et al. [18] both reported that RLA is superior, or at least comparable, to transperitoneal adrenalectomy. The study conducted by Bai et al. [16] only included PHEO patients undergoing transperitoneal adrenalectomy when comparing laparoscopic adrenalectomy to OA. The present study, differing from previous studies, tried to evaluate the perioperative and long-term outcomes of RLA and OA in patients with PHEO larger than 5 cm.

It is important to note that our study has three main limitations. Firstly, this is a retrospective study. Despite adopting PSM to increase the reliability of the conclusions, prospective multicenter studies are still urgently needed to confirm our findings. Secondly, the limited sample size of patients included in this study reduces the reliability of the results to a certain extent. Finally, intraoperative data were collected from electronic anesthetic records that were updated every 5 min; therefore, the accuracy is inadequate and the final conclusions may be affected.

In conclusion, both RLA and OA seem to provide similar perioperative and long-term outcomes for the surgical management of large PHEO, except that RLA is superior to OA in terms of operation time, length of hospital stay, and bowel recovery time. Our results indicate that RLA is an efficacious surgical method which can replace open surgery, to a certain extent, for patients with PHEO larger than 5 cm in diameter.

## Data Availability

The datasets in this study are available from the corresponding author on reasonable request.

## Abbreviations

ASA: Anesthesiologists Physical Status Classification System
BMI: Body mass index
BP: Blood pressure
CI: Confidence interval
HI: Hemodynamic instability
OA: Open adrenalectomy
OR: Odds ratio
PHEO: Pheochromocytoma
PSM: Propensity score matching
RLA: Retroperitoneal laparoscopic adrenalectomy
SD: Standard deviation
vs.: Versus

## References

[1] Lenders JW, Eisenhofer G, Mannelli M, Pacak K (2005). Phaeochromocytoma. Lancet.

[2] Omura M, Saito J, Yamaguchi K, Kakuta Y, Nishikawa T (2004). Prospective study on the prevalence of secondary hypertension among hypertensive patients visiting a general outpatient clinic in Japan. Hypertens Res.

[3] Anderson GJ, Blakeman N, Streeten DH (1994). The effect of age on prevalence of secondary forms of hypertension in 4429 consecutively referred patients. J Hypertens.

[4] Schurmeyer TH, Engeroff B, Dralle H, von Zur MA (1997). Cardiological effects of catecholamine-secreting tumours. Eur J Clin Invest.

[5] Zeiger MA, Thompson GB, Duh QY, Hamrahian AH, Angelos P, Elaraj D, Fishman E, Kharlip J (2009). American Association of Clinical Endocrinologists and American Association of Endocrine Surgeons Medical Guidelines for the Management of Adrenal Incidentalomas: executive summary of recommendations. Endocr Pract.

[6] Gagner M, Lacroix A, Bolte E (1992). Laparoscopic adrenalectomy in Cushing’s syndrome and pheochromocytoma. N Engl J Med.

[7] Hazzan D, Shiloni E, Golijanin D, Jurim O, Gross D, Reissman P (2001). Laparoscopic vs open adrenalectomy for benign adrenal neoplasm. Surg Endosc.

[8] Edwin B, Kazaryan AM, Mala T, Pfeffer PF, Tonnessen TI, Fosse E (2001). Laparoscopic and open surgery for pheochromocytoma. Bmc Surg.

[9] Tiberio GA, Baiocchi GL, Arru L, Agabiti RC, De Ponti S, Matheis A, Rizzoni D, Giulini SM (2008). Prospective randomized comparison of laparoscopic versus open adrenalectomy for sporadic pheochromocytoma. Surg Endosc.

[10] Lang B, Fu B, OuYang JZ, Wang BJ, Zhang GX, Xu K, Zhang J, Wang C, Shi TP, Zhou HX (2008). Retrospective comparison of retroperitoneoscopic versus open adrenalectomy for pheochromocytoma. J Urol.

[11] Autorino R, Bove P, De Sio M, Miano R, Micali S, Cindolo L, Greco F, Nicholas J, Fiori C, Bianchi G (2016). Open versus laparoscopic adrenalectomy for adrenocortical carcinoma: a meta-analysis of surgical and oncological outcomes. Ann Surg Oncol.

[12] Zacharias M, Haese A, Jurczok A, Stolzenburg JU, Fornara P (2006). Transperitoneal laparoscopic adrenalectomy: outline of the preoperative management, surgical approach, and outcome. Eur Urol.

[13] Conzo G, Tartaglia E, Gambardella C, Esposito D, Sciascia V, Mauriello C, Nunziata A, Siciliano G, Izzo G, Cavallo F (2016). Minimally invasive approach for adrenal lesions: systematic review of laparoscopic versus retroperitoneoscopic adrenalectomy and assessment of risk factors for complications. Int J Surg.

[14] Lenders JW, Duh QY, Eisenhofer G, Gimenez-Roqueplo AP, Grebe SK, Murad MH, Naruse M, Pacak K, Young WJ (2014). Pheochromocytoma and paraganglioma: an endocrine society clinical practice guideline. J Clin Endocrinol Metab.

[15] Taffurelli G, Ricci C, Casadei R, Selva S, Minni F (2017). Open adrenalectomy in the era of laparoscopic surgery: a review. Updates Surg.

[16] Bai S, Yao Z, Zhu X, Li Z, Jiang Y, Wang R, Wu B (2019). Comparison of transperitoneal laparoscopic versus open adrenalectomy for large pheochromocytoma: a retrospective propensity score-matched cohort study. Int J Surg.

[17] Chen W, Liang Y, Lin W, Fu GQ, Ma ZW (2018). Surgical management of large adrenal tumors: impact of different laparoscopic approaches and resection methods on perioperative and long-term outcomes. Bmc Urol.

[18] Shiraishi K, Kitahara S, Ito H, Oba K, Ohmi C, Matsuyama H (2019). Transperitoneal versus retroperitoneal laparoscopic adrenalectomy for large pheochromocytoma: comparative outcomes. Int J Urol.

[19] Zhang X, Fu B, Lang B, Zhang J, Xu K, Li HZ, Ma X, Zheng T (2007). Technique of anatomical retroperitoneoscopic adrenalectomy with report of 800 cases. J Urol.

[20] Vorselaars W, Postma EL, Mirallie E, Thiery J, Lustgarten M, Pasternak JD, Bellantone R, Raffaelli M, Fahey TR, Vriens MR (2018). Hemodynamic instability during surgery for pheochromocytoma: comparing the transperitoneal and retroperitoneal approach in a multicenter analysis of 341 patients. Surgery.

[21] Eichhorn-Wharry LI, Talpos GB, Rubinfeld I (2012). Laparoscopic versus open adrenalectomy: another look at outcome using the Clavien classification system. Surgery.

[22] Mercan S, Seven R, Ozarmagan S, Tezelman S (1995). Endoscopic retroperitoneal adrenalectomy. Surgery.

[23] Heger P, Probst P, Huttner FJ, Goossen K, Proctor T, Muller-Stich BP, Strobel O, Buchler MW, Diener MK (2017). Evaluation of open and minimally invasive adrenalectomy: a systematic review and network meta-analysis. World J Surg.

[24] Dudley NE, Harrison BJ (1999). Comparison of open posterior versus transperitoneal laparoscopic adrenalectomy. Br J Surg.

[25] Mohammadi-Fallah MR, Mehdizadeh A, Badalzadeh A, Izadseresht B, Dadkhah N, Barbod A, Babaie M, Hamedanchi S (2013). Comparison of transperitoneal versus retroperitoneal laparoscopic adrenalectomy in a prospective randomized study. J Laparoendosc Adv Surg Tech A.

[26] Raffaelli M, Brunaud L, De Crea C, Hoche G, Oragano L, Bresler L, Bellantone R, Lombardi CP (2014). Synchronous bilateral adrenalectomy for Cushing’s syndrome: laparoscopic versus posterior retroperitoneoscopic versus robotic approach. World J Surg.

[27] Namekawa T, Utsumi T, Kawamura K, Kamiya N, Imamoto T, Takiguchi T, Hashimoto N, Tanaka T, Naya Y, Suzuki H, Ichikawa T (2016). Clinical predictors of prolonged postresection hypotension after laparoscopic adrenalectomy for pheochromocytoma. Surgery.

[28] Pisarska M, Pedziwiatr M, Budzynski A (2016). Perioperative hemodynamic instability in patients undergoing laparoscopic adrenalectomy for pheochromocytoma. Gland Surg.

[29] Scholten A, Vriens MR, Cromheecke GJ, Borel RI, Valk GD (2011). Hemodynamic instability during resection of pheochromocytoma in MEN versus non-MEN patients. Eur J Endocrinol.

